# Perceptions of a healthy diet among Hungarian Roma align with dietary guidelines and are primarily associated with self-perceived eating habits

**DOI:** 10.1038/s41598-026-42171-1

**Published:** 2026-03-09

**Authors:** Anna Kiss, Brigitta Unger-Plasek, Zoltán Lakner, Sándor Soós, Ágoston Temesi

**Affiliations:** 1 Department of Science Policy and Scientometrics, Library and Information Centre, Hungarian Academy of Sciences, Budapest, Hungary; 2 Pro-Sharp Research and Innovation Centre, Budapest, Hungary; 3 Institute of Agricultural and Food Economics, Hungarian University of Agriculture and Life Sciences, Budapest, Hungary; 4 Tashkent State Agrarian University, Тashkent, Uzbekistan; 5 Faculty of Education and Psychology, ELTE Eötvös Loránd University, Budapest, Hungary

**Keywords:** Healthy eating perception, Roma population, Dietary diversity, Ethnic minority, Attitudes to food, Eating behavior, Diseases, Health care, Risk factors

## Abstract

The Roma population is prone to an increased risk for diet-related non-communicable diseases; thus, understanding their perceptions of healthy eating is essential to improve dietary quality and effectively address diet disparities. This study assesses such perceptions among Hungarian Roma and explores their association with sociodemographic variables, weight status, and household dietary diversity. The cross-sectional study included 300 Roma individuals (aged 18 and above) residing in Hungary. The telephone-based survey included questions on perceptions of healthy diet, household dietary diversity, foodways, weight status, and socioeconomic status. Using t-test, one-way ANOVA, linear regression, and chi-square tests, the role of and relationships between variables and perceptions of a healthy diet were examined. Respondents associated healthy eating with a high intake of vegetables and fruits, whole grains, and fresh and natural foods; maintaining a balanced and varied diet; and supporting overall health. Based on the perception of healthy diet (PHD) score, participants’ views generally aligned with a healthy diet. Statistically significant differences were found between perceptions of healthy diet, and sex (*p* = 0.05), and age group (*p* < 0.001). The results of the regression analysis further show that self-perceived healthy eating was the strongest predictor (ß= -0.64, *p* = 0.01) of the PHD score, while sociodemographic factors such as age (ß= -0.46, *p* = 0.008) and sex (ß= 0.21, *p* = 0.07) had more moderate explanatory power. The findings indicate that perceptions regarding healthy diet are not influenced by all socioeconomic factors, BMI, or economic access to food. The findings underscore the need for targeted health-promotion strategies that consider sociocultural factors to promote healthy eating habits and improve health outcomes.

**Supplementary Information:**

The online version contains supplementary material available at 10.1038/s41598-026-42171-1.

## Introduction

Dietary behavior is a key modifiable factor affecting long-term health and overall quality of life. Improving nutrition is a priority in global health policies to reduce the public health burden of non-communicable diseases due to their association with poor dietary quality^1,2^. National dietary guidelines and public health nutrition messages have been promoting healthy eating practices^3,4^, providing evidence-based recommendations tailored to specific national dietary patterns and health needs. However, the adherence to these messages often depends on how they are interpreted by diverse consumer groups. Considering the widespread interest in nutrition and diet, people access related information from various sources. Consequently, consumer perception regarding “healthy” food choices differs significantly.

Several studies have explored people’s perception of “healthy eating”, highlighting diverse perspectives and interpretations of health messages, demonstrating that participants align with the core principles of healthy eating and nutritional guidelines. However, there are significant differences in these perceptions across countries, age groups, employment status, and educational levels^5–9^. Theoretical models of food choice suggest that personal beliefs and values significantly influence food choices^10^. In addition, environmental, social, and cultural factors also play a significant role, reflecting societal norms and group identity^11^. The complex interaction of these factors may influence both dietary intake and dietary behaviors.

Policies have increasingly focused on addressing social health inequalities and promoting healthy diets among vulnerable populations. A scoping review by van der Heijden et al.^12^ examined healthy eating beliefs and behaviors among individuals from low socioeconomic backgrounds, revealing that while these populations generally recognize the importance of healthy eating, their interpretations of what constitutes “healthy” or “good” differ from established definitions.

The Roma are the largest ethnic minority in the European Union, with 10–12 million people, most residing in the Central and Eastern European region^13^. Examining the concept of “healthy diet” and its practical implementation within the Roma community is especially important, considering the challenges affecting their overall health and quality of life. The Roma population is faced with poverty, limited access to education, high rates of unemployment, social exclusion, poor health status, and the resulting short life expectancy^14,15^. In addition, food insecurity – a recurring issue in many Roma communities – alongside cultural, religious, and traditional practices influence their dietary habits, further complicating the understanding and implementation of healthy eating^13–16^. Research indicates that the Roma minority, like other groups from lower socioeconomic backgrounds, often demonstrates less healthy eating behaviors and higher risk of obesity and related health issues^17,18^. Studies have found that dietary intake and food consumption in the Roma population are characterized by poor adherence to nutritional guidelines. Additionally, they follow unhealthy diets, including increased consumption of processed foods high in energy, fat, sugar, and salt, and lower consumption of fruits, vegetables, dairy products, and grains^19–21^.

The Roma constitute the largest ethnic minority in Hungary, representing over 8% of the total population, with demographic trends suggesting further growth in the coming decades^22^. Although the Roma population is internally diverse and heterogeneous, with subgroups differing in cultural traditions, religion, social status, and lifestyle patterns, their residential distribution is characterised by spatial segregation^22,23^. Roma communities are disproportionately concentrated in disadvantaged rural areas, particularly in Northeastern Hungary and Southern Transdanubia^22^. Extensive research has documented the poorer health status of Roma populations compared with the non-Roma majority^24,25^, which is closely linked to their adverse health behavior. Studies show a higher prevalence of chronic non-communicable diseases (e.g., obesity^26,27^, elevated birth and mortality rates, and a significantly greater burden of cardiovascular risk factors among Roma communities, especially those living in socioeconomically deprived settlements^28–30^.

Despite the growing body of evidence on health inequalities, comprehensive research on the dietary intake, eating behaviours, and diet disparities of Roma populations remains limited. Existing studies predominantly reflect the nutritional patterns of Roma living in rural or segregated environments, as a considerable proportion of the Hungarian Roma population resides in such environments. Bárdos et al.^31^ find an unfavorable diet quality among the Hungarian Roma living in settlements compared to the adult general population and a potentially increased risk for diet-related NCDs. Llanaj et al.^19^ demonstrated that the mean daily intake of fat and protein was higher among the Roma than the recommended dietary allowance, originating from a high proportion of animal-based protein and cholesterol intake. Furthermore, Diószegi et al.^32^ found that Roma participants consumed fresh fruits and vegetables less frequently than the general Hungarian population. They also reported a stronger preference for sweet-tasting foods and tended to add greater amounts of sugar to foods and beverages, as well as salting their meals before tasting them more often.

To effectively address these health and nutrition-related challenges, it is essential to contextualize the concept and practice of “healthy diet” by understanding how individuals in minority communities perceive healthy eating. Information on the Roma population’s eating habits and attitudes toward healthy eating remains limited. Accordingly, this study focuses on the perception of healthy eating in a representative sample of adult Roma living in Hungary. It is believed that this is the first study to explore perceptions of healthy diet within the Roma community.

The study addressed the following research questions:

RQ1. How do adult Roma perceive and define a “healthy diet?”

RQ2. How do sociodemographic factors influence their perceptions regarding healthy diets?

RQ3. What is the relationship between the perception of healthy eating among the Roma population and their socioeconomic status, household dietary diversity, and weight status?

## Methods

### Study participants

Data for this cross-sectional study were collected through phone interviews conducted between December 2023 and February 2024. Eligible participants were Roma individuals aged 18 years or older, residing in Hungary, who provided informed consent and clearly indicated that they considered themselves to be of Roma ethnicity. To ensure data accuracy, a screening question identified the household member responsible for food selection and handling, with whom the interview was conducted.

For the telephone interviews, informed consent was obtained verbally as a primary measure, which was subsequently formalized through the documentation process. A standardized verbal consent protocol was implemented at the beginning of each call. At the start of the conversation, the interviewer introduced themselves and explained the purpose of the research, the voluntary nature of participation, and provided the lead researcher’s contact details for any inquiries. Participants were informed that the conversation would be transcribed anonymously, and their data would be handled in accordance with applicable data protection regulations. It was stated that the participant could refuse to answer any question or withdraw from the interview at any time without any negative consequences. After providing this information, the interviewer asked the participant to explicitly state their consent to proceed with the interview. The conversation only continued once this affirmative verbal consent was granted. This verbal consent was formally documented by the interviewer in a dedicated research log for each participant, recording the date, time, and the participant’s explicit agreement to proceed. Consequently, this process transformed the verbal agreement into a written consent within the study documentation, fulfilling the requirement for a verified informed consent. The interviewers assisted participants in understanding each question; the interviews lasted 20–30 min. Overall, 300 Roma people participated in the survey, after providing informed consent.

The participants were recruited using telephone directory randomization, a common and reliable method successfully applied in numerous studies involving hard-to-reach populations^33^. While the participants were randomly selected, quotas were established to ensure that the sample was representative of the Hungarian adult Roma population in terms of age, sex (referring to sex-assigned at birth), and geographical distribution. The demographic structure of their population in Hungary differs significantly from that of non-Roma populations. Accordingly, the sampling was guided by census data on the Roma population provided by the Hungarian Central Statistical Office in 2016, as well as demographic analyses conducted by Hablicsek et al.^34^ (Table 1).

**Table 1:** Geographical distribution of the Roma sample based on population distribution in Hungary.

Region of residence (NUTS-2 regions)	*N*	%
Central Hungary	19	6.3
Central Transdanubia	5	1.7
Western Transdanubia	15	5.0
Southern Transdanubia	65	21.7
Northern Hungary	138	46.0
Northern Great Plain	54	18.0
Southern Great Plain	4	1.3

### Ethics

Participation was anonymous and voluntary; all survey procedures adhered to the General Data Protection Regulation principles. The study was conducted in accordance with the Declaration of Helsinki, and Ethical approval was obtained from the Ethics Committee of the Hungarian University of Agriculture and Life Sciences at the Doctoral School of Economic and Regional Sciences (protocol code 18/2023, November 30, 2023).

### Measure

The questionnaire, based on the research objectives, included sections on perceptions of healthy eating, household dietary diversity, weight status, food security, and socioeconomic information.

### Perception of healthy diet

The participants were asked about their perceptions regarding healthy eating using both closed- and open-ended questions. The items of the closed-ended questionnaire were originally developed by Ferrao et al.^7,35^ and later translated and adapted for use in other countries. In this study, it was translated into Hungarian using back-translation to ensure validity. The adaptation and translation process focused on potential cultural differences that could affect question interpretation. The 12-item perception of healthy diet (PHD) scale is illustrated in Table 2. Respondents’ opinions were measured using a 5-point Likert scale ranging from 1 = strongly disagree to 5 = strongly agree. The internal consistency of PHD scale was assessed using Cronbach’s alpha, resulting in a value of 0.72, indicating acceptable reliability^36^.

**Table 2:** Perceptions regarding healthy diet (from 1 = strongly disagree to 5 = strongly agree, *N* = 300).

Perceptions regarding healthy diet	Percentage of answers according to scale points					Mean (SD)
	1	2	3	4	5	
1. The basis of healthy eating is calorie counting.	26.3	20.3	26.7	10.0	16.7	2.7 (1.3)
2. Avoid consuming foods rich in carbohydrates, sugar, or sweets.	17.7	16.3	32.0	10.7	23.3	3.0 (1.3)
3. Include more fruits and vegetables.	0.3	0.7	3.3	10.7	85.0	4.7 (0.5)
4. Healthy eating should be balanced and varied.	0.7	0.3	3.7	9.0	86.3	4.8 (0.5)
5. We can consume anything but in moderation.	4.7	4.7	17.0	18.0	55.7	4.1 (1.1)
6. Avoid high-fat foods.	13.0	14.0	34.0	15.3	23.7	3.2 (1.3)
7. I believe that following traditions is very important.	10.7	9.3	28.3	18.0	33.7	3.5 (1.3)
8. Healthy foods are tasty.	3.0	6.0	30.3	20.7	40.0	3.8 (1.1)
9. Whether a food is healthy depends on an individual’s specific needs.	4.0	9.7	28.3	21.3	36.7	3.7 (1.1)
10. Healthy eating involves consuming nutrient-rich foods.	3.3	8.3	25.0	27.0	36.3	3.8 (1.1)
11. Healthy eating involves consumption of fresh and natural foods.	1.7	1.7	11.0	23.3	62.3	4.4 (0.8)
12. Healthy eating plays an important role in maintaining good health.	0.0	0.7	2.7	13.0	83.7	4.8 (0.5)

Alongside the objective assessment of the perception of healthy diet, three open-ended questions were included to gauge participants’ understanding and associations of “healthy eating”:

- What does eating mean to you?
- What do you understand by healthy eating?
- How would you describe your personal diet?

### Household dietary diversity

The Household Dietary Diversity Score (HDDS) is a widely used indicator of economic access to food that measures household access to various foods. Developed as a survey-based indicator, it quantifies the range of food items consumed by a household within the last 24 h^37^. The HDDS score was determined based on 12 food groups, aligning with those used to construct the FAO food balance sheets. Each food group was assigned a score of 1 if any item was consumed and 0 otherwise. There is no universal cutoff point; therefore, for this study, it was determined based on Swindale and Bilinsky’s^37^ framework. A higher total score reflects greater household capacity to access food.

### Weight status

Weight status was assessed through self-reported height and weight data. General weight status and classification into specific categories were based on BMI according to WHO criteria^38^. It was calculated using the formula: BMI = weight (kg)/[height (m)]^2^, and categorized into four groups according to the conventional WHO classification.

### Socioeconomic data

Socioeconomic data variables included sex, age, living environment, income, education, marital status, family size, and occupation. Relative income poverty and material deprivation was considered based on the At Risk of Poverty or Social Exclusion indicator^39^. Material deprivation refers to financial hardship and limited access to basic goods. Participants were classified as severely deprived if they experienced at least four in nine specified items of material deprivation listed in the questionnaire. Accordingly, participants were classified into three socioeconomic status categories.

### Data analysis

#### Statistical analysis

All respondents who agreed to participate provided complete answers to all closed-ended questions. Basic descriptive statistical methods were employed to summarize the main characteristics of the dataset. One of the main variables was the values regarding the perception of healthy eating scale, from which a score was generated for statistical analysis. The perception of healthy eating scale was adjusted to calculate an average score without Point 3 on the Likert scale (3 = neither agree nor disagree), affecting the results. The scale items were transformed as follows: -2 (totally disagree), -1 (disagree), 0 (neutral), 1 (agree), and 2 (strongly agree). They were then averaged for each participant reflecting their perceptions regarding healthy eating. Items 1, 2, 6, and 7 were reversed before calculating the average. Following the methodology of Ferrão et al.^7,35^, the final scores ranged from − 2 to + 2 and were interpreted as: -2 to -1.5 (not at all compliant with healthy eating), -1.5 to -0.5 (not compliant), -0.5 to 0.5 (poor compliance), 0.5 to 1.5 (partially compliant), and 1.5 to 2 (fully compliant).

The normality of the data distribution was assessed with the Kolmogorov-Smirnov test. The results indicated that the data followed a normal distribution (*p* > 0.05), allowing for the application of parametric tests. Student’s t-test, one-way ANOVA, linear regression, and chi-square tests were used to examine the role of and relationships between sociodemographic variables and perceptions of a healthy diet, and between anthropometric variables, HDDS, and self-perceived eating habits. Student’s t-test for independent samples was used to compare the means of two groups, while one-way ANOVA compared the means of three or more groups. For post-hoc analysis following ANOVA, Tukey’s HSD (honestly significant difference) test was applied to evaluate differences between groups. Eta-squared (η^2^) was used in ANOVA to measure the proportion of variance explained by the independent variables, with effect size ranges 0.01 for small, 0.06 for medium, and 0.14 for large effect. In the t-test, Cohen’s d was applied to measure the standardized difference between two means; the thresholds are 0.2 for small, 0.5 for medium, and 0.8 for large effect. We conducted a multivariate linear regression analysis in which the PHD score served as the dependent (outcome) variable. In the assessment of the model, we focused on the effect size measure (beta values) in order to evaluate the contribution of variables in explaining the PHD score. We also run an assumption check for the model, especially we analyzed the collinearity of predictors to avoid bias in the model parameters. According to the VIF values, no multicollinearity was detected among the predictor variables (VIF < 5). The chi-square test was used to assess the associations between certain variables, with Cramer’s V applied in some cases to assess the strength of the association between two categorical variables. The effect size ranges for Cramer’s V are 0.07 for small, 0.21 for medium, and 0.35 for a large effect. A significance level of 5% (*p* < 0.05) was applied across all tests. Statistical analysis was performed using Jamovi (version 2.6) statistical software^40^.

#### Qualitative data analysis

The responses to the open-ended questions were analyzed using thematic analysis. The analysis followed the standard six-step procedure introduced by Braun and Clarke^41^. First, all responses were read repeatedly to familiarize with the data. Eight of the 300 participants provided non-substantive responses, indicating an inability to give a meaningful answer. These responses still carry interpretive value, as they may reflect uncertainty or difficulty in verbalizing personal experiences with the topic. These instances were documented but excluded from the subsequent thematic analysis. In the second step, initial codes were generated inductively to capture meaningful units of information related to participants’ perceptions. These codes were then organized into broader patterns, forming preliminary themes. In the fourth step, the initial themes were reviewed and refined, ensuring that they accurately represented the data and formed coherent, distinct thematic categories. Next, the themes were clearly defined and named, reflecting their core meaning. Finally, the themes were summarized and interpreted in relation to the research questions. Throughout the analytical process, two researchers coded the data independently, and disagreements were resolved through discussion to enhance the credibility of the result.

## Results

The sample included 300 Hungarian Roma adults; females accounted for 53.3% and males 46.7%; average age was M = 57 (standard deviation (SD) = 12.8), from 18 to 81. Furthermore, 12.3% were single, 66.0% were married/living with a partner; 61.7% lived in rural areas, 31.0% in urban areas. The geographical distribution of the sample reflects the national distribution of the Roma population in Hungary, with 46% of respondents residing in Northern Hungary, 21.7% in Southern Transdanubia, and 18.0% in the Northern Great Plain (Table 1). Among respondents, 40.7% had a vocational secondary school education, and 20% completed high school. 30.7% of participants were classified as normal weight, 36% as overweight, and 29% as obese; the average BMI was 28.3 (SD = 6.25). Table 3 summarizes the sociodemographic and other characteristics of the sample.

**Table 3:** The sociodemographic and other characteristics of the sample (*N* = 300).

Sociodemographic variables	Variable levels	Frequency (percentage %)	Women	Men
			160 (53.3)	140 (46.7)
Age	18 ≤ age ≤ 44	47 (15.7)	19 (6.3)	28 (9.3)
	45 ≤ age ≤ 59	142 (47.3)	74 (24.7)	68 (22.7)
	60 ≤ age ≤ 75	93 (31.0)	56 (18.7)	37 (12.3)
	Older than 75	18 (6.0)	11 (3.7)	7 (2.3)
Education level	Primary school	43 (14.3)	29 (9.7)	14 (4.7)
	Vocational secondary school	112 (40.7)	52 (17.3)	70 (23.3)
	High school	58 (19.3)	32 (10.7)	26 (8.7)
	University degree	77 (25.7)	47 (15.7)	30 (10.0)
Marital status	Single	37 (12.3)	12 (4.0)	25 (8.3)
	Married/living together	198 (66.0)	100 (33.3)	98 (32.7)
	Divorced	30 (10.0)	9 (3.0)	21 (7.0)
	Widow	35 (11.7)	27 (9.0)	8 (2.7)
Professional status	Employed	189 (63.0)	93 (31.0)	96 (32.0)
	Unemployed	15 (5.0)	11 (3.7)	4 (1.3)
	Retired	96 (32.0)	56 (18.7)	40 (13.3)
Living environment	Rural	185 (61.7)	104 (34.7)	81 (27.0)
	Urban	93 (31.0)	42 (14.0)	51 (17.0)
	Province seat/capital city	22 (7.3)	14 (4.7)	8 (2.7)
Socioeconomic status	Low	140 (46.7)	83 (27.7)	57 (19.0)
	Low-moderate	83 (27.7)	38 (12.7)	45 (15.0)
	Moderate-high	77 (25.7)	39 (13.0)	38 (12.7)
BMI categories	Normal weight (18.50 ≤ BMI ≤ 24.99)	92 (30.7)	55 (18.3)	37 (12.3)
	Overweight (25.00 ≤ BMI ≤ 29.99)	108 (36.0)	56 (18.7)	52 (17.3)
	Obesity I. (30.00 ≤ BMI ≤ 34.99)	87 (29.0)	40 (13.3)	47 (15.7)
	Obesity II. (≥ 35.00)	13 (4.3)	9 (3.0)	4 (1.3)
Household dietary diversity score	Low (≤ 7)	182 (40.0)	100 (33.3)	82 (27.3)
	Adequate (meeting the reference value (≥ 7.1))	118 (60.0)	60 (20.0)	58 (19.3)
My eating patterns are healthy	Strongly disagree	16 (5.3)	10 (3.3)	6 (2.0)
	Disagree	23 (7.7)	9 (3.0)	14 (4.7)
	Neither agree nor disagree	110 (36.6)	58 (19.3)	52 (17.3)
	Agree	94 (31.3)	48 (16.0)	46 (15.3)
	Strongly agree	57 (19.0)	35 (11.7)	22 (7.3)

### Definitions of healthy eating

**Fig. 1:**
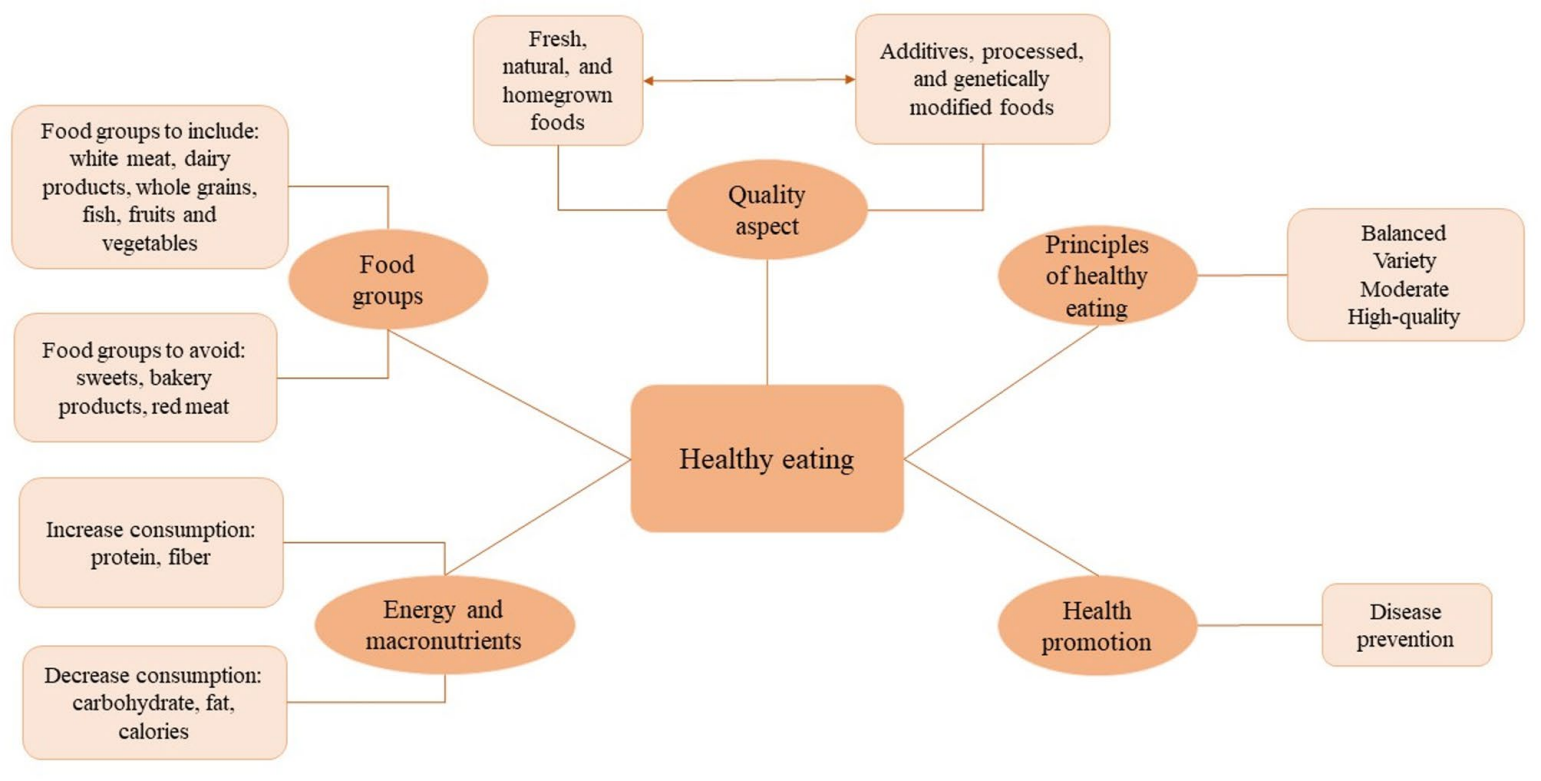
Themes emerged in the definition of healthy eating. (source: author’s own editing)

The respondents frequently used specific terms such as nutrients, food groups, key principles, and health promotion in relation to healthy eating (Fig. 1). A key theme was the importance of consuming plenty of fruits and vegetables, mentioned by every second respondent, as a fundamental component of a healthy diet.

Participant 79: “To consume as many vegetables and fruits as possible.”

Participant 152: “.eating smaller portions, with half of the meal comprising vegetables.”

Other significant themes included reducing carbohydrates and foods high in sugar (mentioned by 10.3% of respondents) and the importance of incorporating more grains and fiber (mentioned by 10%). Limiting the consumption of sweets and bakery products, particularly bread, among grain products was also highlighted.

Participant 81: “One should avoid eating flour-based products and bread, and reduce the consumption of sugary drinks and pastries.”

One-fifth of respondents identified healthy eating as maintaining a balanced and varied diet, practicing moderation, and prioritizing high-quality foods. Additionally, many respondents emphasized the importance of fresh, natural, and homegrown foods, while avoiding additives, processed, and genetically modified foods (9.6%).

Participant 35: “.buy products with known ingredients, or those that are homegrown, like vegetables.”

Some respondents mentioned the importance of adequate daily protein intake, recognizing meat as a key component of a healthy diet. They distinguished between white and red meat, with 10% suggesting reducing red meat consumption, and 15% considering white meat as healthy. Meanwhile, 6% referenced specific guidelines, such as recommending 3–5 meals a day, to maintain a healthy eating routine. The use of terms such as protein, fiber, and vitamins indicates that respondents recognize key nutritional concepts related to health and nutrient needs. Overall, while some aspects of their definitions of healthy eating aligned with current dietary recommendations and guidelines, others—such as high meat consumption—did not. They indicated that certain foods—such as fruits and vegetables—should be increased in the diet, while others—like processed foods and foods high in fat—should be reduced or avoided.

In summary, respondents described healthy eating as consuming a well-balanced diet with moderate energy intake rich in vegetables, fruits, white meat or dairy products, and whole-grain foods.

Participant 117: “At least half of the diet should include fruits and vegetables, complemented by some meat and a small amount of bread, eliminating sugar. Alcohol intake should be minimal, a slightly higher consumption of dairy is recommended, and genetically modified foods should be avoided.”

Participant 278: “One should avoid overeating and be mindful to their food choices, including consuming vegetables, fruits, and an adequate amount of protein.”

### Perceptions about healthy diet

Participants’ perceptions generally aligned with the healthy diet defined by the PHD scale (Table 2). Consensus was higher on certain items, including consuming fruits and vegetables (85% strongly agreed), maintaining a balanced and varied diet (86.3% strongly agreed), consuming fresh and natural foods (62.3% strongly agreed), and recognizing how a healthy diet helps maintain health (83.7% strongly agreed). However, the responses varied for items such as consumption of carbohydrate-rich foods, and role of tradition in healthy eating patterns. A similar pattern emerged in participants’ perception of healthiness of food groups (Supplementary file 1).

Table 4 illustrates the group differences between sociodemographic characteristics and perceptions of a healthy diet. A minor but significant difference was observed between sexes (*p* = 0.05). Both women and men had consistent perceptions regarding a healthy diet; however, women had a higher score (0.91 ± 0.32) compared to men (0.84 ± 0.31). The effect size, as indicated by Cohen’s d = 0.227, reflects a minor difference between the groups. There was no significant differences by educational level in perceptions regarding healthy diet.

A significant difference was observed in perceptions of healthy diet among different age groups (*p*-value = 0.001) with a moderate effect size (η² = 0.057). Senior adults had the highest mean score (0.97 ± 0.30), followed by young adults (0.93 ± 0.32). The mean scores across different marital statuses ranged between 0.5 and 1.5, indicating that single, married/living together, divorced, and widowed participants held perceptions aligning with a healthy diet. Meanwhile, retired participants had a higher score (0.93 ± 0.30) compared to employed participants (0.85 ± 0.32), but the difference was not statistically significant.

**Table 4:** Group differences along sociodemographic characteristics and perceptions of a healthy diet.

Variable	Variable levels	Mean ± SD	*P*-value and effect size
Sex	Men	0.84 ± 0.31	*P* = 0.051 Cohen’s d = 0.227
	Women	0.91 ± 0.32	
Age	18 ≤ age ≤ 44	0.93 ± 0.32	*P* < 0.001^a^ η2 = 0.057
	45 ≤ age ≤ 59	0.80 ± 0.32	
	60 ≤ age ≤ 75	0.97 ± 0.30	
	Older than 75	0.88 ± 0.34	
Education level	Primary school	0.95 ± 0.31	*P* = 0.085 η2 = 0.022
	Vocational secondary school	0.88 ± 0.32	
	High school	0.90 ± 0.29	
	University degree	0.80 ± 0.34	
Marital status	Single	0.87 ± 0.36	*P* = 0.297 η2 = 0.019
	Married/living together	0.87 ± 0.31	
	Divorced	0.82 ± 0.35	
	Widow	0.97 ± 0.31	
Professional status	Employed	0.85 ± 0.32	*P* = 0.135 η2 = 0.013
	Unemployed (including homemaker)	0.89 ± 0.38	
	Retired	0.93 ± 0.30	
Socioeconomic status	Low	0.91 ± 0.31	*P* = 0.138 η2 = 0.013
	Low-moderate	0.83 ± 0.34	
	Moderate-high	0.85 ± 0.31	
Living environment	Rural	0.87 ± 0.34	*P* = 0.168 η2 = 0.008
	Urban	0.85 ± 0.29	
	County seat/capital city	0.97 ± 0.24	

ANOVA was applied for the comparison of three or more groups. Student’s t-test was used for independent samples to compare the two groups (level of significance: 5%).

^a^Significant differences between the first, second, and third groups.

The mean healthy diet score values for each BMI group range from 0.82 (overweight) to 1.00 (obesity II.), with no significant differences between them (*p* = 0.08). All BMI groups had perceptions aligned with a healthy diet. Participants’ self-reported perceptions of whether their eating patterns are healthy were also analyzed. There was a significant difference in responses (*p* = 0.004) with a moderate effect size (η^2^ = 0.050). Those who strongly disagreed with the statement, “My eating patterns are healthy” had the highest mean score in the analysis. In terms of HDDS, no significant difference was found; however, participants whose economic access to food was better had a higher overall score than those with a lower HDDS (Table 5).

**Table 5:** Group differences along BMI, HDDS, and perceptions of healthfulness of eating patterns and healthy diet.

Variable		Mean ± SD	*P*-value and effect size
BMI	Normal weight (18.50 ≤ BMI ≤ 24.99)	0.92 ± 0.33	*P* = 0.08 η2 = 0.023
	Overweight (25.00 ≤ BMI ≤ 29.99)	0.82 ± 0.32	
	Obesity I. (30.00 ≤ BMI ≤ 34.99)	0.88 ± 0.32	
	Obesity II. (≥ 35.00)	1.00 ± 0.15	
My eating patterns are healthy	Strongly disagree	1.01 ± 0.34	*P* = 0.004^a^ η2 = 0.050
	Disagree	0.85 ± 0.44	
	Neither agree nor disagree	0.81 ± 0.33	
	Agree	0.86 ± 0.28	
	Strongly agree	0.99 ± 0.26	
HDDS	Low	0.86 ± 0.33	*P* = 0.384 Cohen’s d = 0.103
	Adequate	0.90 ± 0.30	

ANOVA was applied for the comparison of three or more groups. Student’s t-test was used for independent samples to compare the two groups (level of significance 5%).

^a^Significant difference between the third and fifth group.

The regression model shows a low explanatory power (adjusted R^2^ = 0.09), indicating that although the included variables collectively have a statistically significant effect (*p* < 0.001), they explain only a modest proportion of the variance in the PHD score (Table 6). The effect sizes of the individual predictors (beta coefficients) are generally small, suggesting weak associations between most variables and the PHD score (Table 6), which is consistent with the results of the variance analysis. Among the sociodemographic variables, age (with a negative association across categories: ß = -0.46, *p* = 0.008) and sex (ß = 0.21, *p* = 0.07) showed limited influence, while the self-perception that “my eating patterns are healthy” emerged as the strongest predictor in the model (ß = -0.64, *p* = 0.01). Overall, these findings suggest that sociodemographic characteristics are not the most influential factors of the PHD score in this model.

**Table 6:** Results of the linear regression analysis predicting the PHD score.

Predictor	Coefficient (B)	SE	T	*p*	Beta
Interceptᵃ	1.06166	0.1095	9.69166	< 0.001	
Living environment					
Rural	Ref.				
Urban	− 0.0271	0.0409	− 0.66183	0.509	− 0.08363
Capital city	0.08741	0.0728	1.20051	0.231	0.26978
Age categories					
18 ≤ age ≤ 44	Ref.				
45 ≤ age ≤ 59	− 0.15145	0.0564	− 2.68312	0.008	− 0.46742
60 ≤ age ≤ 75	0.02983	0.0791	0.3772	0.706	0.09207
Older than 75	− 0.04327	0.1161	− 0.37251	0.71	− 0.13353
Education level					
Primary school	Ref.				
Vocational secondary school	0.04729	0.0623	0.75911	0.448	0.14595
High school	0.03781	0.0686	0.55139	0.582	0.1167
University degree	− 0.07925	0.0692	− 1.14476	0.253	− 0.2446
Marital status					
Single	Ref.				
Married/living together	0.00758	0.0596	0.12711	0.899	0.02339
Divorced	− 0.06822	0.0811	− 0.84162	0.401	− 0.21055
Widow	4.87E−04	0.0859	0.00568	0.995	0.0015
Professional status					
Employed	Ref.				
Unemployed	− 0.06103	0.09	− 0.67774	0.499	− 0.18834
Retired	− 0.03663	0.0665	− 0.55118	0.582	− 0.11305
Gender					
Men	Ref.				
Women	0.07042	0.0388	1.81579	0.07	0.21735
BMI categories					
Normal weight	Ref.				
Overweight	− 0.04957	0.047	− 1.0539	0.293	− 0.153
Obesity I.	− 0.0028	0.0496	− 0.05637	0.955	− 0.00863
Obesity II.	0.06807	0.0948	0.71773	0.474	0.21009
Socioeconomic status					
Low	Ref.				
Low-moderate	− 0.03986	0.0498	− 0.80002	0.424	− 0.12302
Moderate-high	0.0184	0.0543	0.33875	0.735	0.05678
Household dietary diversity score					
Low	Ref.				
Adequate	0.01973	0.0385	0.51217	0.609	0.06089
My eating patterns are healthy					
Strongly disagree	Ref.				
Disagree	− 0.13228	0.1042	− 1.26968	0.205	− 0.40826
Neither agree nor disagree	− 0.21052	0.0864	− 2.43735	0.015	− 0.64972
Agree	− 0.12463	0.0876	− 1.42221	0.156	− 0.38465
Strongly agree	− 0.00939	0.0902	− 0.10408	0.917	− 0.02899

ᵃRepresents reference level.

Participants’ self-assessment of their eating patterns varied across socioeconomic status groups. Table 7 illustrates the relationship between socioeconomic status and participants’ perceptions of how healthy their eating patterns are, revealing a significantly different pattern of distribution (χ^2^= 24.9, *p* = 0.02). The findings suggest a positive association between higher socioeconomic status and perceived dietary healthiness, although a majority of participants in all groups remained neutral. Cramer’s coefficient indicated that this association was moderate (V = 0.204). The association between perceptions of healthy eating patterns and BMI categories was examined using chi-square analysis. There was no significant difference (χ^2^=17.8, *p* = 0.12), but a larger percentage of participants in the normal weight BMI group agreed or strongly agreed with the statement “My eating patterns are healthy” (56.5%) compared to those in the obese BMI I and II categories (Table 7).

**Table 7:** Self-perceived healthiness of eating patterns across socioeconomic and weight status.

	My eating patterns are healthy					
	Strongly disagree	Disagree	Neither agree nor disagree	Agree		Strongly agree
Socioeconomic status *N* (%)						
Low	12 (8.6)	17 (12.1)	52 (37.1)	31 (22.1)		28 (20)
Low-Moderate	1 (1.2)	4 (4.8)	32 (38.6)	27 (32.5)		19 (22.9)
Moderate-High	3 (3.9)	2 (2.6)	26 (33.8)	36 (46.8)		10 (13)
χ^2^= 24.9, *p* = 0.02						
BMI categories, N (%)						
Normal	9 (9.8)	5 (5.4)	26 (28.3)	29 (31.5)	23 (25.0)	
Overweight	3 (2.8)	9 (8.3)	38 (35.2)	41 (38.0)	17 (15.7)	
Obese I	3 (3.4)	7 (8.0)	40 (46.0)	21 (24.1)	16 (18.4)	
Obese II	1 (7.7)	2 (15.4)	6 (46.2)	3 (23.1)	1 (7.7)	

χ^2^ = 17.8, *p* = 0.12.

## Discussion

This study examined how Hungarian Roma perceive and define healthy eating, and explored their associations with sociodemographic variables, body mass index (BMI), and household dietary diversity. Based on the healthy diet perception score, participants’ views generally aligned with a healthy diet. Statistically significant differences were found between perceptions of healthy diet and sex, and age group. In contrast, no significant differences were found across other sociodemographic variables (socioeconomic status, living environment, marital status, or employment status), nor across BMI or HDDS categories in the analysis of variance. A clear association was found between the PHD score and participants’ self-assessment of the healthiness of their own eating habits. Regression analysis further confirmed that self-perceived healthy eating was the strongest predictor of the PHD score, while age and sex showed statistically significant but only modest explanatory effects. Overall, these findings suggest that sociodemographic characteristics play a limited role in shaping the PHD score within this model.

Analysis of the open-ended responses indicated that respondents primarily defined healthy eating as a high intake of vegetables and fruits, whole grains, and fresh, natural foods, as well as maintaining a balanced and varied diet to support overall health. Several themes emerged in the associations of healthy eating, including food quality aspects, general principles of healthy eating, health promotion, and foods and nutrients considered either beneficial or to be avoided. Among these, regular consumption of fruits and vegetables and adequate protein intake, mainly from meat and dairy products, were the most frequently mentioned components. In general, the participants’ responses largely reflected the key messages of established dietary guidelines. However, certain areas, particularly the intake of proteins and carbohydrates, appeared to be less clearly understood and were more difficult for participants to assess the role of these nutrients and foods that contain them within a healthy diet.

The results of the subjective assessment demonstrate that many Roma respondents linked healthy eating to a high intake of vegetables and fruits, higher intake of meat, consumption of whole grains, fresh and natural foods, maintaining a balanced and varied diet, and health promotion. This perception is reflected in messages that are commonly emphasized in health promotion campaigns and nutritional guidelines. The findings align with other studies and show that participants were broadly aware of healthy eating recommendations^42^. While the findings are only partially comparable to previous studies – most of which focused on different target groups – one of the key findings of this study is that the perception of healthy eating among a representative sample of the Roma population aligns with that of higher socioeconomic groups in other studies.

The most fundamental element of participants’ definition of healthy eating was a high intake of vegetables and fruits. This may stem from the fact that emphasizing fruit and vegetable consumption has been a long-standing cornerstone of Hungary’s food-based dietary guidelines^43^. This result aligns with other studies. Paquette^10^ found that regardless of age, consuming large quantities of vegetables and fruits was most frequently mentioned as part of a healthy diet. Furthermore, many Roma participants believed that higher meat consumption was healthy. Paquette’s^10^ literature review presented mixed findings regarding the role of meat, with some studies reporting that people perceive limiting meat consumption as healthy, and others perceiving higher meat consumption as healthy. An important factor behind higher meat consumption among Roma communities lies in their cultural traditions, where meat symbolizes wealth^44^. Traditional Roma dishes, many of which are rich in meat, such as stuffed cabbage, remain an essential part of the diet of Hungarian Roma. Among wealthier families, frequent meat consumption and generous portion sizes remain central features, reflecting the cultural value and significance of meat in conveying well-being^44^. In contrast, meat dishes consumed by poorer Roma families are dominated by those made from cheaper, bony cuts (such as duck wing roots) or offal. Other studies also highlight the cultural significance of meat within Roma dietary practices. Olišarová et al.^45^ highlighted the central role of meat in characterizing the Roma diet, while Kozubik’s study similarly reported that meat constituted a fundamental component of the diet among wealthier Roma families. Furthermore, Olišarová et al.^45^ and Kozubik et al.^46^ demonstrated that eating is a symbol of social status within the Roma community.

In this study, participants struggled to assess the healthiness of grains, sugar, and salt. Reducing the intake of carbohydrates and sweets emerged as a common theme in the context of healthy eating, yet responses varied when assessed through objective measurements. This may be related to the fact that Roma cuisine is characterized by a distinctive flavour profile in which intense flavours are particularly prominent. Furthermore, food rich in carbohydrates, kneaded dough dishes, and sweet pastries have become integrated into Roma culinary traditions^44^. Diószegi et al. showed that Roma participants exhibited a stronger preference for sweet tastes, including higher added sugar use and more frequent pre-tasting salting of meals. The authors suggest that these patterns may be influenced by broader environmental and contextual factors, including food availability and cultural norms^32^.

Although the Roma participants in this research had a perception of what constitutes a healthy diet , studies suggest that the dietary intake of Hungarian Roma does not meet nutritional recommendations^19,31,32^. Llanaj et al.^19^ reported that fat and protein intake among Hungarian Roma exceeds recommendations, largely due to high consumption of animal-based foods. Earlier studies (e.g., Diószegi et al.) also indicate lower fruit and vegetable intake and a preference for sweeter and more heavily seasoned foods^32^. Consistent with this, Bárdos et al. found generally poor adherence to dietary guidelines in the Hungarian Roma populations living in segregated colonies^31^. Studies in other countries examining the diet and food consumption of the Roma population have shown that their diet is lower in fruits, vegetables, and whole grains, but rich in fats, saturated fats, and salt content compared to non-Roma population^20,21^. These dietary patterns are characteristic of the European Roma population. The findings highlight that while respondents largely reflect health promotion messages that are likely to reach the Roma population, they do not reflect in their food consumption and dietary intake. This strengthens the evidence that health promotion strategies alone may not lead to behavioral change, and there is a need for approaches that consider sociocultural factors.

Regarding the objective measure, participants demonstrated accurate perceptions of a healthy diet, and statistically significant associations were observed between these perceptions and certain sociodemographic variables (sex and age group). Young adults (aged 18–44) and senior adults (aged 60–75) scored the highest, a finding inconsistent with Ferrão et al.^7^ and Boustani and Guiné^8^. The fact that older respondents had higher scores may be attributed to their greater likelihood of making food choices based on health considerations and reporting stronger intentions and behaviors toward healthy eating^47^. Women scored higher than men, aligning with Ferrão et al.^7^, Ismail et al.^9^, and Boustani and Guiné^8^, although not all these studies found a significant difference between sexes. These differences in perception may be attributed to variations in women’s food choices, greater involvement in food preparation, and stronger beliefs regarding the importance of healthy eating^16,48^. Roma women hold a unique role in food preparation, where the act of cooking acts as a primary means of expressing love. Moreover, their culinary skill directly influences their level of respect they receive within their communities [44]. Consequently, Roma women may present an ideal target group for future nutritional education and interventions, as they directly influence their families’ dietary practices.

There were no significant differences in healthy diet perception scores according to education level, marital status, socioeconomic status, profession, or living environment. Meanwhile, those living in county seats/capital city and rural areas had higher healthy diet perception scores than their urban counterparts. This finding aligns with Ferrão et al.^7^, yet appears surprising given that neighborhood deprivation has been associated with lower dietary quality and higher obesity rates^49,50^. Additionally, there was no significant correlation between BMI and healthy diet perception scores. Regardless of BMI category, participants’ perceptions remained consistent with a healthy diet, suggesting that irrespective of BMI, Roma individuals nevertheless acknowledge what constitutes a healthy diet.

The results indicate that perceptions regarding healthy diet are not dependent on all socioeconomic characteristics, serving as an important foundation for public health programs. This suggests that the main barrier to healthy eating may not be Roma people’s low socioeconomic positions or limited exposure to nutrition messages, but rather sociocultural factors. According to Olišarová et al.^45^, the Roma nutritional culture is characterized by moderate overconsumption, and eating symbolizes social status. Van der Heijden et al.^12^ also emphasized the importance of understanding complex beliefs and sociocultural and economic influences on healthy eating in low socioeconomic populations. Interventions aimed at improving dietary quality should focus on these factors in the Roma population.

### Limitations

This study has certain limitations worth noting to better contextualize the findings. The sample was not representative in terms of educational background. While national statistics indicate that the proportion of Roma individuals in Hungary with higher education is relatively low, approximately 25% of respondents reported having completed higher education, potentially introducing a certain bias. Nevertheless, the findings can be considered valuable, as the sample was representative in terms of sex, age, and geographical distribution. A further limitation of the study is that it did not assess participants’ health status or diet-related chronic disease during the selection process. These factors can influence an individual’s perception of a healthy diet. Consequently, the sample may have included participants with specific dietary requirements or health-related constraints, which could have potentially biased the reported perceptions of a healthy diet. Future research should consider incorporating health and diet-related screening questions to explore how these factors differentiate perceptions of healthy eating.

This study’s cross-sectional design is known for the limitation that no causality may be concluded from the results; however, cross-sectional designs are optimal for examining larger populations and for identifying patterns that can inform the development of future longitudinal or intervention studies. In addition, the use of self-reported measures (e.g., dietary perceptions, body weight) may introduce recall or social desirability bias. To mitigate potential misreporting, we employed trained interviewers and included open-ended questions throughout the survey, which allowed for consistency checks and the identification of conflicting or implausible responses during data cleaning.

The nature of the applied methodology limited the ability to capture deeply embedded sociocultural norms related to nutrition. Consequently, some relevant factors influencing dietary behavior were not measured in the present study, which introduces the possibility of residual confounding. Such underlying norms may play an important role in shaping eating practices and should be explored in future research using qualitative methods such as focus group discussions or in-depth individual interviews. Selection bias should also be considered, as the survey specifically targeted individuals responsible for food purchasing and preparation. As a result, younger adults (aged 18–25) were somewhat underrepresented, since they may not yet be primarily responsible for these tasks. In addition, women were slightly overrepresented in the sample, reflecting traditional gender roles in food-related responsibilities. However, this did not substantially affect the representativeness. It is believed that no such study has been conducted in the general Hungarian (non-Roma) population. Therefore, the findings cannot be compared across different ethnic groups in Hungary. Future studies involving participants from different ethnic groups are needed.

### New contribution to the literature

It is believed to be the first study to assess perceptions of healthy diet among Roma population using a representative sample in terms of sex, age, and geographical distribution. This study integrates perceptions of healthy diet with factors such as dietary diversity, socioeconomic, and weight status to understand the relationship between these factors. Perceptions generally aligned with a healthy diet, associating it with a high consumption of fruits, vegetables, whole grains, fresh foods, and balanced meals. Among the analyzed sociodemographic characteristics, age and sex showed statistically significant but only modest explanatory effects on the PHD score. This suggests a limited role for sociodemographic characteristics in shaping the PHD score, with sociocultural factors likely holding a stronger influence on the perception of healthy eating in the Hungarian Roma population. These findings help gain a deeper understanding of how Roma people perceive and define healthy eating, which is essential for promoting and implementing strategies to encourage healthier eating behaviour and address diet disparities. The findings also underscore the need for culturally-sensitive and community-focused health promotion strategies addressing not only dietary awareness but also the broader social and cultural contexts where food choices are made.

## Supplementary Information

Below is the link to the electronic supplementary material.

Supplementary Material 1

## Data Availability

The data that support the findings of this study are not openly available due to reasons of sensitivity and are available from the corresponding author upon reasonable request.
