# Supplementary material for: Perceptions of a healthy diet among Hungarian Roma align with dietary guidelines and are primarily associated with self-perceived eating habits
Source: Sci Rep. 2026 Mar 9;16:12784. doi: 10.1038/s41598-026-42171-1 (PMC13096374; doi:10.1038/s41598-026-42171-1)
Supplement: Supplementary file 1 — Supplementary Material 1 [file 41598_2026_42171_MOESM1_ESM.docx]

Perceptions of the healthiness of food groups

Most participants considered vegetables (98%), fruits (95.3%), fish (92.7%), eggs (88.3%), and white meat (85.7%) as healthy. Additionally, 65% and 59.3% of respondents classified sweet and soft drinks as unhealthy, respectively, while perceptions surrounding grains, red meat, fats, and oils varied across categories, with one-third classifying these items as "neither healthy nor unhealthy" (Supplementary Table 1).

Supplementary Table 1. Perceptions of the healthiness of food groups

| Food groups | **Unhealthy (%)** | **Neither healthy nor unhealthy (%)** | **Healthy**  **(%)** |
| --- | --- | --- | --- |
| Grains | 17.0 | 38.0 | 45.0 |
| Fruits | 1.0 | 3.7 | 95.3 |
| Vegetables | 0.3 | 1.7 | 98.0 |
| Milk and dairy products | 6.0 | 18.3 | 75.7 |
| Pork and beef | 16.3 | 29.3 | 54.3 |
| Poultry | 2.3 | 12.0 | 85.7 |
| Vegetable oil | 21.3 | 26.3 | 52.3 |
| Fats | 21.7 | 24.3 | 54.0 |
| Eggs | 2.0 | 9.7 | 88.3 |
| Fish | 3.7 | 3.7 | 92.7 |
| Sugar, salt | 37.3 | 34.7 | 28.0 |
| Sweets | 65.0 | 21.3 | 13.7 |
| Soft drinks | 59.3 | 27.0 | 13.7 |
